# Association of Lithium Exposure With Alzheimer’s Disease: A Propensity Score-Matched Cohort Study

**DOI:** 10.7759/cureus.107597

**Published:** 2026-04-23

**Authors:** Anand Deonarine, Rawan Elkomi, Mekdem Bisrat, Syed Fahad Gillani, Chukwudalu Ononenyi, Rana Elkomi, Miriam B Michael, Nicholas Azinge

**Affiliations:** 1 Internal Medicine, Howard University Hospital, Washington, DC, USA; 2 Internal Medicine, King Edward Medical University, Lahore, PAK; 3 Internal Medicine, University of Maryland, Baltimore, USA

**Keywords:** alzheimer’s disease, bipolar disorder, dementia risk, lithium, neuroprotective effects

## Abstract

Background: Lithium has been widely studied for its potential neuroprotective effects through mechanisms implicated in Alzheimer’s disease (AD), yet evidence from large real-world populations remains inconsistent. Clarifying the association between lithium exposure and AD risk in routine clinical practice is important given lithium’s widespread use in mood disorders.

Objective: This study aims to examine the association between lithium exposure and the incidence and time to diagnosis of AD among adults with bipolar disorder or recurrent major depressive disorder.

Methods: We conducted a retrospective cohort study using the TriNetX Global Collaborative Network. Adults aged 50 years or older with bipolar disorder or recurrent major depressive disorder and no prior diagnosis of dementia or neurodegenerative disease were identified. Patients with documented lithium exposure for at least one year were propensity score-matched 1:1 to patients without lithium exposure on demographic and clinical covariates. Follow-up began one year after cohort entry. The primary outcome was incident AD, identified using the International Classification of Diseases, 10th Revision, code G30. Cumulative risk measures, Kaplan-Meier survival analysis, and Cox proportional hazards models were used to assess associations.

Results: The matched cohorts included 67,020 patients each. Incident AD occurred in 34 patients in the lithium cohort and 69 patients in the no lithium cohort. Lithium exposure was associated with a lower recorded risk of AD, with a risk ratio of 0.49 (95% confidence interval (CI) = 0.33-0.74) and an odds ratio of 0.49 (95% CI = 0.33-0.74). Time-to-event analysis demonstrated a lower hazard of AD diagnosis among lithium-exposed patients (hazard ratio = 0.48; 95% CI = 0.32-0.73; log rank p < 0.001). Among patients who developed AD, the number of AD-coded encounters did not differ significantly between the cohorts.

Conclusion: In this large real-world cohort of older adults with mood disorders, lithium exposure was associated with a lower recorded incidence and hazard of AD. Although absolute event rates were low, the consistency of findings across analytic approaches supports a potential protective association and highlights the need for prospective studies with detailed exposure and cognitive assessments.

## Introduction

Alzheimer's disease (AD) is a progressive neurodegenerative disorder and the leading cause of dementia, representing a significant global public health challenge [1]. Despite extensive research, effective treatments to prevent or reverse the disease remain limited, though emerging therapeutic classes show promising potential. Consequently, there is growing interest in repurposing existing medications with known neuroprotective properties. Lithium, a mood stabilizer used in the long-term treatment of bipolar disorder, has long been a candidate for this purpose due to its established biological mechanisms.

Preclinical and laboratory studies have consistently demonstrated lithium's potential neuroprotective effects. It is a potent inhibitor of glycogen synthase kinase-3 beta (GSK-3β), an enzyme implicated in both amyloid-beta plaque formation and tau hyperphosphorylation, two of the key pathological hallmarks of AD [2,3]. Other research has shown that lithium may promote neurogenesis and clear toxic protein aggregates, further supporting its role as a potential disease-modifying agent [4]. A recent study using mouse models even suggested that lithium depletion in the brain could be a precursor to the disease itself, indicating a fundamental neuroprotective role [5].

However, the findings from large-scale human observational studies on lithium and dementia risk have been inconsistent. While some population-based analyses have shown a protective effect, suggesting that lithium use is associated with a lower risk of dementia, other studies have found no significant association or, in some cases, a higher risk [6]. These discrepancies highlight the complex interplay of confounding factors that are difficult to control in a real-world clinical setting. Specifically, patients on long-term lithium therapy often have severe underlying conditions, such as bipolar disorder, which may independently increase their risk of dementia, a phenomenon known as confounding by indication.

Given the conflicting evidence and the potential for confounding factors, this study aims to clarify the real-world association between long-term lithium exposure and the risk of AD. Using a large, demographically diverse patient cohort from the TriNetX Global Collaborative Network, we conducted a propensity score-matched analysis to create comparable patient groups [7]. Our primary objective was to evaluate the association between long-term lithium exposure and the incidence of AD, while our secondary objective was to explore this association for early-onset and late-onset forms of the disease.

## Materials and methods

Study design and data source

We conducted a retrospective cohort study using the TriNetX Global Collaborative Network, a federated electronic health record database comprising deidentified longitudinal data from participating healthcare organizations. Available data include demographics, diagnoses, medications, procedures, and clinical encounters. All analyses were performed within the TriNetX Analytics Platform in February 2026. Because all data were deidentified and aggregated, institutional review board approval was not required.

Cohort selection

Eligible patients were adults aged 50 years or older with a documented diagnosis of bipolar disorder or recurrent major depressive disorder, identified using International Classification of Diseases, 10th Revision (ICD-10), codes F31 and F33, respectively. Two cohorts were constructed based on lithium exposure status.

The lithium-exposed cohort included patients with evidence of lithium use identified using medication and procedure codes, including RxNorm 42351, ATC code N05AN, VA medication class CN750, or Current Procedural Terminology code 80178. Lithium exposure was defined as any recorded lithium prescription or therapeutic drug monitoring code occurring at or after cohort entry. The comparison cohort consisted of patients with bipolar disorder or recurrent major depressive disorder and no documented lithium exposure at any point in the electronic health record.

Patients were excluded if they had any diagnosis of dementia or neurodegenerative disease prior to cohort entry, including AD (ICD-10 G30), vascular dementia (F01), dementia in other diseases classified elsewhere (F02), unspecified dementia (F03), amnestic disorders due to known physiological conditions (F04), or other degenerative diseases of the nervous system (G31-G32). The index date was defined as the earliest date on which all inclusion and exclusion criteria were met.

Matching and covariate adjustment

To reduce confounding, 1:1 propensity score matching was performed between patients exposed to lithium and those not exposed using nearest neighbor matching within the TriNetX Analytics Platform. The propensity score model included demographic variables (age at index, sex, race, and ethnicity) and clinical covariates selected a priori based on relevance to cognitive outcomes and lithium prescribing.

Clinical covariates included hypertensive diseases (ICD-10 I10-I1A), diabetes mellitus (E08-E13), thyroid disorders (E03, E05), overweight and obesity (E66), schizophrenia spectrum and other non-mood psychotic disorders (F20-F29), and psychotropic medication use. Medication covariates included antipsychotics (ATC N05A), anticonvulsants (VA class CN400), antidepressants (ATC N06A), and sedative-hypnotic agents.

After matching, 67,020 patients remained in each cohort. Covariate balance was assessed using standardized mean differences as reported by the platform, with values below 0.1 indicating adequate balance.

Follow-up periods and outcomes

Follow-up began 365 days after the index date to reduce misclassification of preexisting disease and reverse causation. Patients were followed until the first occurrence of the outcome of interest, the end of the available observation window, or the last recorded clinical encounter, whichever occurred first. Maximum follow-up extended to approximately five years after the index.

The primary outcome was incident AD, identified using the ICD-10 code G30. Outcomes were assessed consistently across cumulative incidence, Kaplan-Meier survival, and number-of-instances analyses. Median survival time was not reached in either cohort during the follow-up period; therefore, survival probabilities at the end of the observation window were reported.

Statistical analysis

The cumulative risk of AD was calculated for each cohort. Measures of association included risk difference (RD), risk ratio (RR), and odds ratio (OR), each with corresponding 95% confidence intervals (CIs). Time-to-event analyses were conducted using Kaplan-Meier survival curves, with between-group comparisons assessed using the log-rank test. Cox proportional hazards regression models were used to estimate hazard ratios (HRs) with 95% CIs, and proportional hazard assumptions were evaluated within the analytic platform.

A number-of-instances analysis was performed to assess the frequency of AD-related diagnostic encounters among patients who developed AD. This analysis excluded patients with zero outcome instances by design and compared mean counts between cohorts using two-sample t-tests. Mean values, standard deviations (SDs), and medians were reported as generated by the TriNetX platform. All statistical tests were two-sided, and statistical significance was defined as a p value less than 0.05.

## Results

Primary outcome: AD (G30)

Incident AD occurred in 34 of 67,020 patients in the lithium cohort and 69 of 67,020 patients in the no-lithium cohort (risk 0.001 in both cohorts as displayed by the platform). The absolute RD was -0.001 (95% CI = -0.001 to 0.000; z = -3.450; p = 0.001). Relative measures showed a lower recorded AD risk in the lithium cohort, with an RR of 0.493 (95% CI = 0.327-0.743) and an OR of 0.492 (95% CI = 0.327=0.743) (see Table 1).

**Table 1:** Risk and time to diagnosis for AD outcomes AD: Alzheimer's disease; CI: confidence interval; RD: risk difference; RR: risk ratio; OR: odds ratio; HR: hazard ratio

Outcome	Risk (lithium vs. no lithium)	RD (95% CI)	RR (95% CI)	OR (95% CI)	Log-rank test (χ^2^)	HR (95% CI)
AD overall (G30)	0.001 vs. 0.001	-0.001 (-0.001 to -0.000)	0.493 (0.327-0.743)	0.492 (0.327-0.743)	12.559 (p < 0.001)	0.484 (0.321-0.729)

Time-to-event analyses

Kaplan-Meier survival analysis demonstrated survival probabilities at the end of the time window of 99.92% for the lithium cohort and 99.83% for the no-lithium cohort; median survival was not reached in either cohort. The log-rank test showed a significant difference between the cohorts (χ² = 12.559; df = 1; p < 0.001). In Cox proportional hazards modeling, lithium exposure was associated with a lower hazard of AD diagnosis (HR = 0.484; 95% CI = 0.321-0.729). The proportional hazard assumption test was not significant (χ² = 0.319; df = 1; p = 0.572).

Number-of-instances analyses (per visit)

Among patients with at least one AD-coded instance, the mean number of instances per patient was 2.559 (SD = 2.776; median = 1.5) in the lithium cohort vs. 3.362 (SD = 4.768; median = 2) in the no-lithium cohort. This difference was not statistically significant (t = -0.908; df = 101; p = 0.366), suggesting no meaningful difference in postdiagnosis documentation frequency between the cohorts.

Table 1 presents cumulative risk estimates, absolute and relative measures of association, and time-to-event analyses for incident AD in the propensity score-matched lithium-exposed and non-lithium-exposed cohorts. Reported measures include RD, RR, OR, and HR with corresponding 95% CIs. Time-to-event differences were assessed using Kaplan-Meier survival analysis and the log-rank test. Although absolute risk values appear similar due to rounding, relative and time-to-event measures indicate a lower observed incidence of AD in the lithium-exposed cohort.

## Discussion

In this large, propensity score-matched retrospective cohort study, lithium exposure was associated with a lower recorded incidence and hazard of AD among adults with bipolar disorder or recurrent major depressive disorder. Across cumulative risk measures and time-to-event analyses, lithium-treated patients demonstrated approximately half the relative risk of AD compared with matched patients without lithium exposure. Although absolute event rates were low in both cohorts, the direction of association was consistent across analytic approaches.

The observed association is biologically plausible in light of lithium's established molecular effects relevant to AD pathophysiology. Lithium inhibits GSK-3β, a kinase involved in amyloid beta production and tau phosphorylation, processes central to AD pathology [8]. Experimental work has shown that modulation of this pathway can alter amyloid processing and tau-related neurotoxicity, providing a mechanistic framework through which lithium could influence neurodegenerative risk. Forlenza et al. further demonstrated that long-term lithium treatment in amnestic mild cognitive impairment was associated with disease-modifying properties, supporting the translational relevance of GSK-3β inhibition in humans [9].

Beyond amyloid and tau signaling, lithium has been shown to influence neuronal survival and plasticity in experimental models. Preclinical studies demonstrate effects on synaptic function, neurogenesis, and cellular resilience under neurodegenerative stress conditions [10]. Lithium has also been shown to enhance autophagy pathways in dopaminergic cells, suggesting broader neuroprotective mechanisms that extend across neurodegenerative contexts [11]. These findings suggest that lithium's neurobiological effects extend beyond mood stabilization and may intersect with pathways relevant to cognitive decline.

Observational studies examining lithium exposure and dementia outcomes in humans have yielded mixed results. Some population-based analyses report a lower incidence of dementia among lithium-treated patients, particularly with sustained exposure [12]. Other studies have found no significant association when varying exposure definitions or analytic frameworks are applied [13]. A recent systematic review and meta-analysis examining lithium use in bipolar disorder similarly found heterogeneous effects on dementia risk, highlighting the sensitivity of findings to study design and population characteristics [14]. Early randomized evidence from a multicenter lithium trial in AD also demonstrated the complexity of translating preclinical signals into clinical benefit [15]. These inconsistencies underscore the sensitivity of observational findings to cohort construction, exposure modeling, and outcome ascertainment.

Methodological considerations are particularly relevant in the context of psychiatric populations. Patients with bipolar disorder demonstrate measurable cognitive differences compared with the general population, independent of treatment exposure [16]. These baseline differences may influence both the timing and likelihood of cognitive diagnoses in routine clinical care. As a result, careful cohort matching and analytic strategies are required to isolate associations attributable to medication exposure rather than underlying disease characteristics.

The present findings add to this literature by demonstrating a consistent association between lithium exposure and lower recorded AD incidence in a large, demographically diverse real-world cohort. The consistency across cumulative risk, relative measures, and time-to-event analyses supports the internal coherence of the observed signal. At the same time, the heterogeneity of prior findings highlights the need for continued evaluation across datasets, study designs, and outcome definitions.

Interpretation of observational associations must remain cautious. As with all nonrandomized analyses, measured relationships reflect complex interactions between treatment selection, disease severity, and healthcare utilization patterns [17]. Understanding how these factors shape observed dementia risk estimates remains an important area for future investigation, particularly given the modifiable risk factor burden identified in recent commission reports [18].

Clinical implications

These findings contribute important real-world evidence to an ongoing debate regarding lithium and neurodegenerative risk. In a large, well-matched cohort of older adults with bipolar disorder or recurrent major depressive disorder, lithium exposure was associated with a lower recorded incidence and hazard of AD. This association was observed consistently across cumulative risk and time-to-event analyses.

From a clinical perspective, these results may provide reassurance regarding the long-term cognitive safety of lithium in appropriately selected patients. Lithium remains a cornerstone therapy for mood stabilization and suicide risk reduction, and concerns regarding potential cognitive harm have contributed to hesitancy in its use among older adults. The present findings suggest that, in routine clinical practice, lithium exposure is not associated with an increased risk of AD and may be associated with a lower recorded incidence.

At the same time, these results should not be interpreted as evidence to initiate lithium for the purpose of dementia prevention. Clinical decision-making should continue to prioritize psychiatric indications, patient tolerance, renal and thyroid monitoring, and individualized risk-benefit assessment.

Limitations

This study has several limitations inherent to its retrospective observational design. Although propensity score matching was used to balance measured demographic and clinical covariates between lithium-exposed and non-lithium cohorts, residual confounding cannot be fully excluded. In particular, unmeasured factors such as duration and severity of mood disorder, educational attainment, family history of dementia, and genetic risk factors, including APOE genotype, were not available in structured electronic health record data.

Lithium exposure was defined using medication and laboratory codes in the electronic health record, which may not accurately reflect adherence, cumulative dose, serum lithium levels, or duration of continuous treatment. As a result, dose-response relationships and threshold effects could not be evaluated. Similarly, outcome ascertainment relied on diagnostic coding for AD, which may vary across health systems and clinicians and may be influenced by differences in healthcare utilization or access to specialty evaluation.

The number of observed AD events was low relative to the overall cohort size, reflecting the rarity of recorded AD diagnoses within the available follow-up window. While this is consistent with prior real-world studies of neurodegenerative outcomes, rare event settings can increase sensitivity to outcome misclassification. In addition, competing risks, such as mortality, were not explicitly modeled and may influence estimates of time to diagnosis.

Finally, this analysis was conducted within a federated electronic health record network and reflects recorded clinical practice rather than standardized research assessments. As such, findings should be interpreted as associations observed in routine care rather than causal effects.

## Conclusions

In this large propensity score-matched retrospective cohort study, lithium exposure was associated with a lower recorded incidence and hazard of AD among adults with bipolar disorder or recurrent major depressive disorder. Although absolute event rates were low, the direction and consistency of the association across analytic approaches support the robustness of the observed signal. These findings align with mechanistic and preclinical evidence suggesting potential neuroprotective effects of lithium and add real-world clinical context to a heterogeneous body of observational literature. Further research, including prospective studies and analyses incorporating treatment duration, serum levels, and standardized cognitive outcomes, is needed to clarify the relationship between lithium exposure and neurodegenerative risk. Taken together, this study supports the continued careful use of lithium in appropriate patients and highlights the value of large-scale real-world data in addressing clinically relevant questions at the intersection of psychiatry and neurodegeneration.
